# Dynamic Cerebral Autoregulation Is Acutely Impaired during Maximal Apnoea in Trained Divers

**DOI:** 10.1371/journal.pone.0087598

**Published:** 2014-02-03

**Authors:** Troy J. Cross, Justin J. Kavanagh, Toni Breskovic, Bruce D. Johnson, Zeljko Dujic

**Affiliations:** 1 Griffith Health Institute and Heart Foundation Research Centre, Griffith University, Gold Coast Campus, Queensland, Australia; 2 Department of Physiology, University of Split School of Medicine, Split, Croatia; 3 Division of Cardiovascular Diseases, Mayo Clinic, Rochester, Minnesota, United States of America; Georgia State University, United States of America

## Abstract

**Aims:**

To examine whether dynamic cerebral autoregulation is acutely impaired during maximal voluntary apnoea in trained divers.

**Methods:**

Mean arterial pressure (MAP), cerebral blood flow-velocity (CBFV) and end-tidal partial pressures of O_2_ and CO_2_ (PETO_2_ and PETCO_2_) were measured in eleven trained, male apnoea divers (28±2 yr; 182±2 cm, 76±7 kg) during maximal “dry” breath holding. Dynamic cerebral autoregulation was assessed by determining the strength of phase synchronisation between MAP and CBFV during maximal apnoea.

**Results:**

The strength of phase synchronisation between MAP and CBFV increased from rest until the end of maximal voluntary apnoea (*P*<0.05), suggesting that dynamic cerebral autoregulation had weakened by the apnoea breakpoint. The magnitude of impairment in dynamic cerebral autoregulation was strongly, and positively related to the rise in PETCO_2_ observed during maximal breath holding (*R* ^2^ = 0.67, *P*<0.05). Interestingly, the impairment in dynamic cerebral autoregulation was not related to the fall in PETO_2_ induced by apnoea (*R* ^2^ = 0.01, *P = *0.75).

**Conclusions:**

This study is the first to report that dynamic cerebral autoregulation is acutely impaired in trained divers performing maximal voluntary apnoea. Furthermore, our data suggest that the impaired autoregulatory response is related to the change in PETCO_2_, but not PETO_2_, during maximal apnoea in trained divers.

## Introduction

Cerebral autoregulation is a compensatory mechanism which acts to maintain cerebral blood flow at a near constant value, despite fluctuations in arterial blood pressure or intracranial pressure [1], [2]. It can be argued that autoregulation of cerebral blood flow and, by extension, cerebral O_2_ delivery is nowhere more important for human survival than during prolonged asphyxia. And perhaps an extreme model of asphyxia can be found in the breath-hold diver performing a maximal apnoea [3], [4].

In trained breath-hold divers, arterial O_2_ tension may approach <30 mmHg, and CO_2_ tension may reach as high as 91 mmHg, by the breakpoint of maximal apnoea [5]–[9]. In spite of this extreme asphyxia, cerebral tissue oxygenation is well-maintained during maximal apnoea due to a compensatory cerebral hyperaemia [10]. The majority of the rise in cerebral blood flow during apnoea is achieved by vasodilation of cerebral resistance vessels, consequent to hypercapnia [11], [12]. Maximal apnoea also evokes a powerful vasoconstriction of the peripheral tissues, leading to a pronounced arterial hypertension [10], [13], [14]. Indeed, approximately one-third of the cerebral blood flow response to brief apnoea (i.e., 20 s) is contributed by the increase in arterial blood pressure [11]. The observation that arterial hypertension partly explains the rise in cerebral blood flow suggests that cerebral autoregulation is functionally impaired during maximal voluntary apnoea. To date, no study has examined dynamic cerebral autoregulation (dCA) during prolonged breath holding.

The primary aim of this study was to quantify the effectiveness of dCA during maximal “dry” apnoea in trained breath-hold divers. Previous studies indicate that the relationship between arterial CO_2_ on cerebrovascular pressure-reactivity is nonlinear, primarily affecting the phase more so than the amplitude dynamics of the cerebral autoregulatory response [15]–[18]. Moreover, it is well-established that arterial O_2_ tension exerts a modulatory effect on dCA, wherein arterial hypoxaemia seemingly impairs the autoregulatory response [19]–[21]. Consequently, dCA was assessed using phase synchronisation analysis [22], [23] on spontaneous, beat-to-beat values of mean arterial blood pressure (MAP) and middle cerebral artery blood flow-velocity (CBFV) during maximal apnoea. It was hypothesised that trained divers would display signs of impaired dCA by the breakpoint of apnoea, evidenced by a significant increase in the strength of phase synchronisation between arterial pressure and cerebral blood flow-velocity. Moreover, it was expected that those divers with the highest levels of arterial hypercapnia would demonstrate the largest increases in phase synchronisation by the apnoea breakpoint.

## Methods

### Ethics Statement

The present study conformed to the principles outlined in the Declaration of Helsinki and was approved by the research ethics board at the School of Medicine, University of Split, Croatia.

### Subjects and Experimental Design

Eleven trained, male apnoea divers (28±2 yr; 182±2 cm, 76±7 kg) volunteered to participate in the present study, and provided written informed consent. Within the 6 months prior to experimental testing, the divers were engaged in apnoea training at least 3 times a week, were each session lasted approximately 60 to 90 min. The subjects underwent a pre-participatory health screening to ensure they were physically active non-smokers, with no history of cardiac, pulmonary or metabolic disease.

Subjects first performed a number of pulmonary function tests in the supine position, after which they were instructed to remain supine for 10 min before performing breath-hold manoeuvres (i.e., rest period). Breath-hold manoeuvres commenced after full inflation to total lung capacity (TLC) while wearing a nose-clip. The subjects were instructed to keep their glottis closed during each breath-hold. Subjects were discouraged from performing preparatory hyperventilation, and were not allowed to perform glossopharyngeal insufflations prior to the manoeuvre. The subjects completed two practice trials, followed by 3 experimental breath holds, separated by at least 10 min of supine rest. A repeat apnoea trial did not begin until haemodynamic variables had returned to baseline. Subjects were encouraged to completely relax their respiratory musculature during the early period of the breath-hold (i.e., easy-going phase) and, should the urge arise, to allow respiratory contractions to develop “*naturally”* toward the end of breath holding. The subjects were also instructed to avoid activation of their peripheral musculature. No further explanation of the apnoeic effort was provided.

### Pulmonary Function Testing and End-tidal Gases

Before each experiment, the subjects performed a forced vital capacity (FVC) manoeuvre while in the supine posture (Quark PFT, Cosmed, Rome, Italy). All pulmonary function testing was performed and reported in accordance with the American Thoracic Society guidelines [24]. Expired gases were continuously sampled at the mouth using a mass spectrometer (AMIS 2000, Innovision A/S, Odense, Denmark) and, from these data, the end-tidal partial pressures of O_2_ and CO_2_ (PETO_2_ and PETCO_2_) were computed.

### Electrocardiogram, Arterial Oxygen Saturation, Blood Pressure and Cerebral Blood Flow-velocity

Arterial O_2_ saturation of haemoglobin (SaO_2_) was measured via finger pulse oximetry (Poet II, Criticare Systems, Waukesha, WI). Beat-by-beat arterial blood pressure was measured using a pneumatic cuff placed around the middle phalanx of the non-dominant hand, and was connected to a photoplethysmograph (Finometer, Finapress Medical Systems, Arnhem, Netherlands). The hand bearing the finger cuff was positioned at the level of the heart in order to negate hydrostatic pressure artifact. The hand was kept in this position for the duration of the experimental protocol. Heart rate and rhythm were monitored continuously using a standard 3-lead electrocardiogram module that was interfaced with the Finometer unit. CBFV was obtained via transcranial Doppler ultrasound of the proximal middle cerebral artery, measured using a 2-MHz probe fixed at a constant angle over the right posterior temporal “window” (Transcranial Doppler, Multigon, Yonkers, NY, USA).

### Data Collection and Signal Processing

All signals were sampled continuously at 1000 Hz (Powerlab 16 SP, ADInstruments Inc., Castle Hill, Australia) and stored on a personal computer for off-line analyses. Mean arterial pressure (MAP) and mean CBFV were computed as the arithmetic mean of the arterial pulse wave and transcranial Doppler flow-velocity signals over each cardiac beat interval, respectively [25]. Cerebrovascular resistance index (CVRi) was calculated as MAP/CBFV. The discontinuous, beat-by-beat time series were resampled to 2 Hz using cubic spline interpolation.

### Phase Synchronisation and Dynamic Cerebral Autoregulation

Phase synchronisation analysis describes the strength of interaction between the intrinsic phases (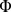) of two weakly-coupled, nonstationary, nonlinear chaotic oscillators [26], [27]. Two oscillators are said to be ‘synchronised’ or ‘phase-locked’ when the difference in their phases remain constant over a given length of time [22], [23], [28], [29]. In the present study, the strength of phase synchronisation between MAP and CBFV was used to assess the effectiveness of dCA during maximal apnoea. That is, MAP and CBFV were considered to act as two autonomous oscillators, weakly-coupled in their phases through the action of the effector, dCA. According to this paradigm, dCA is intact when the oscillators CBFV and MAP are not synchronised in their phase dynamics [22], [23]; i.e., the oscillator CBFV operates near-independently of MAP. Conversely, when the dynamics of MAP and CBFV are tuned such that their phases become synchronised, fluctuations in arterial pressure are “passively” transmitted through the cerebral vasculature unabated, portending autoregulatory failure.

The strength of phase coupling between MAP and CBFV, and thus the effectiveness of dCA, was quantified using the phase synchronisation index (PhSI). The method used to calculate PhSI was modified from that described by others [22], [23], [30]. In brief, the 2 Hz interpolated data for MAP and CBFV were band-pass filtered using a 5^th^ order, zero-phase Butterworth filter, with a low and high cut-off frequency of 0.01 and 0.15 Hz, respectively. These frequency cut offs were chosen based on previous observations that dCA is most active within the low to very-low frequency region [i.e., 0.01–0.15 Hz; 1,2]. This filtering approach also had the effect of removing variability in each time-series due to respiration and the cardiac cycle. The intrinsic phases of the filtered MAP and CBFV time series, 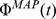 and 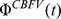, were extracted using the Hilbert transform [23], [30]. The distribution of phase differences between MAP and CBFV was then determined as: 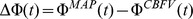. 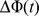 was wrapped to the interval –180 to 180°, such that a negative value suggests that changes in MAP ‘lead’ those observed in CBFV. PhSI is a quantity that represents the time-dependent stability of 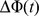, and was calculated as:
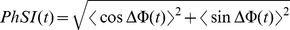
(1)where the brackets 〈•〉 denotes a 30-s moving average window. PhSI lies within the interval 0≤PhSI ≤1. A value of PhSI = 1 indicates perfect phase synchronisation, while PhSI = 0 suggests complete absence of phase synchronisation between signals MAP and CBFV. Therefore, in the context of the present study, a decrease in PhSI during apnoea was taken to represent an increased effectiveness of dCA, whereas an increase in PhSI was interpreted as an impairment of autoregulatory processes while breath holding. Phase synchronisation analysis was performed using MATLAB® (The MathWorks, Inc., Natick, USA).

### Statistical Analyses

Data were averaged across repeated trials. The dependent variables were averaged into four time-epochs representing: the resting period, the first and last 30-s of maximal apnoea, and the final 30-s of the 2 min recovery period. PETO_2_ and PETCO_2_ could not be measured while subjects were breath holding. As such, PETO_2_ and PETCO_2_ data were organised into 4 bins representing: 1) the resting period; 2) the mean of values observed 30-s immediately prior to apnoea; 3) the minimum and maximum values of PETO_2_ and PETCO_2_, respectively, within the first 3 breaths immediately following the apnoea breakpoint; and: 4) the final 30-s of the 2-min post-apnoea recovery period. One-way analyses of variance were used to evaluate the effect of these time epochs on cardiovascular and cerebrovascular variables, and on parameters derived the phase synchronisation analysis. Pair-wise comparisons were assessed using the *Bonferroni* post-hoc adjustment. Pearson’s correlation coefficient was used to determine whether any change in dCA function, assessed via PhSI, could be related to the changes in either PETO_2_ and PETCO_2_ observed during maximal voluntary apnoea. Results are presented as mean ± standard error of the mean (SEM) unless stated otherwise. All data were analysed using SPSS 20.0 (SPSS, Inc., Chicago, IL, USA). Statistical analyses were considered significant if *P*<0.05.

## Results

### Pulmonary Function and Breath Holding Performance

All subjects presented with normal pulmonary function, where FVC, forced expiratory volume in 1 s (FEV_1_) and FEV_1_/FVC were 6.30±0.41 L (116±7% predicted), 5.14±0.24 L (113±5% predicted) and 0.82±0.02 (100±2% predicted). The average duration of maximal voluntary apnoea was 199±11 s (range 154 to 261 s). The coefficient of variation computed for the subject’s repeated apnoeic efforts was 7±2%.

### Expired Gases, Cardiovascular and Cerebrovascular Variables during Maximal Voluntary Apnoea

During the resting period before apnoea, PETO_2_ and PETCO_2_ were 121±3 mmHg and 41±1 mmHg, respectively. In the 30-s period before apnoea, PETO_2_ increased to 131±3 mmHg (*P*<0.05) while PETCO_2_ fell to 38±1 mmHg (*P*<0.05). Thus, despite verbal instruction, the subjects hyperventilated (albeit mildly) before performing maximal apnoea. Immediately post-apnoea, PETO_2_ and PETCO_2_ were 61±4 mmHg and 56±1 mmHg – both values were different from those observed at rest (*P*<0.05). The average changes in PETO_2_ and PETCO_2_ from rest until the apnoea breakpoint were –57±7 mmHg and +14±1 mmHg, respectively. By the end of the 2-min recovery period, PETO_2_ and PETCO_2_ remained different from rest (104±5 mmHg and 46±4 mmHg, respectively; both *P*-values were <0.05).

**Figure 1:**
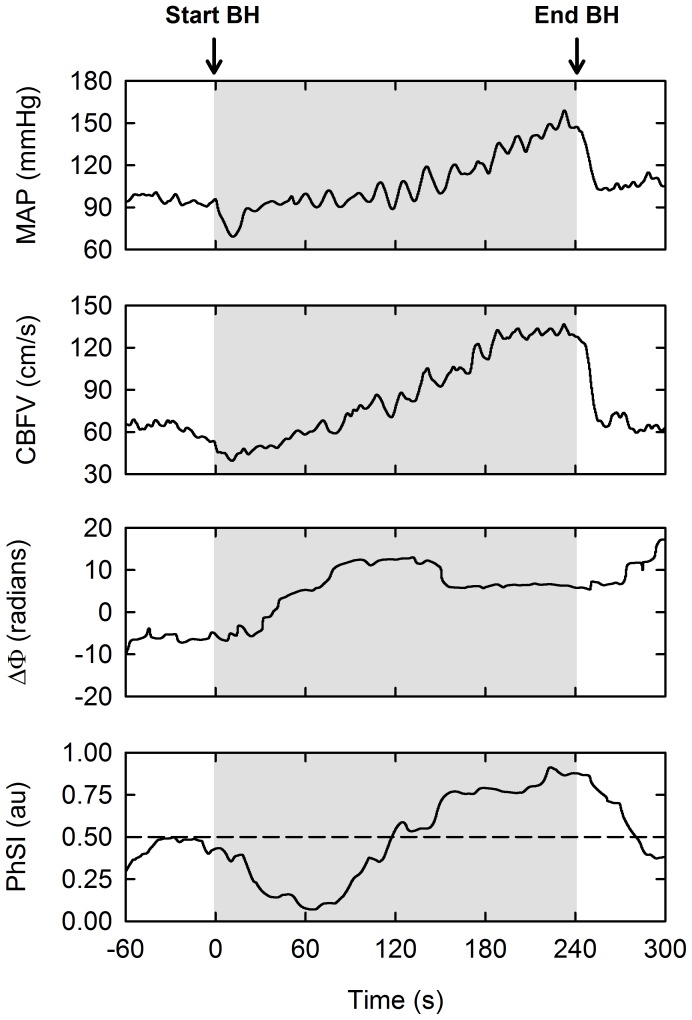
Individual data from a representative subject during maximal voluntary apnoea. BH: breath-hold; MAP: mean arterial pressure; CBFV: cerebral blood flow-velocity; 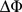: phase angle between the MAP and CBFV time series; PhSI: phase synchronisation index. N.B.: values for 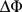 and PhSI were obtained from the low-frequency region of the MAP–CBFV relationship (i.e., 0.01–0.15 Hz), where dynamic cerebral autoregulation is considered most active.

**Figure 1** displays the changes in MAP and CBFV during maximal apnoea for a representative trained diver. The time-course changes in cardiovascular and cerebrovascular variables during maximal apnoea are reported in **Table 1**. CVRi increased from rest to the beginning of maximal apnoea (*P*<0.05). Conversely, MAP and CBFV decreased over the same period of time (*P*<0.05). Thereafter, MAP and CBFV progressively increased (*P*<0.05), while CVRi and SaO_2_ continuously decreased (*P*<0.05), from the beginning until the end of apnoea. After 2-min of recovery, the above parameters returned to baseline. Apart from an initial rise at the beginning of apnoea (*P*<0.05), HR was not different from rest during and after breath holding.

**Table 1:** Cardiovascular and cerebrovascular parameters before, during and after maximal voluntary apnoea.

	Rest	Maximal apnoea		Δ_End–Rest_	Post
		Start	End		
HR (beats•min^-1^)	72±2	94±8*	71±4†	–1±4	81±4
MAP (mmHg)	92±4	67±4*	123±4* †	31±3	96±4†
CBFV (cm•s^-1^)	51±3	35±2*	93±9* †	42±8	52±4†
CVRi (units)	2.1±0.2	2.3±0.1*	1.6±0.1* †	–0.6±0.2	2.2±0.2†
SaO_2_ (%)	99±1	98±1	87±2* †	–12±2	99±1

Values represent means ± SEM from 11 divers. HR: heart rate; MAP: mean arterial pressure; CBFV: middle cerebral artery blood flow-velocity; CVRi: cerebrovascular resistance index in units of mmHg•cm^-1^•s; SaO_2_: arterial haemoglobin oxygenation.

* *P*<0.05 compared with resting values.

† *P*<0.05 compared with previous time-point.

### Dynamic Cerebral Autoregulation during Maximal Voluntary Apnoea

**Figure 2:**
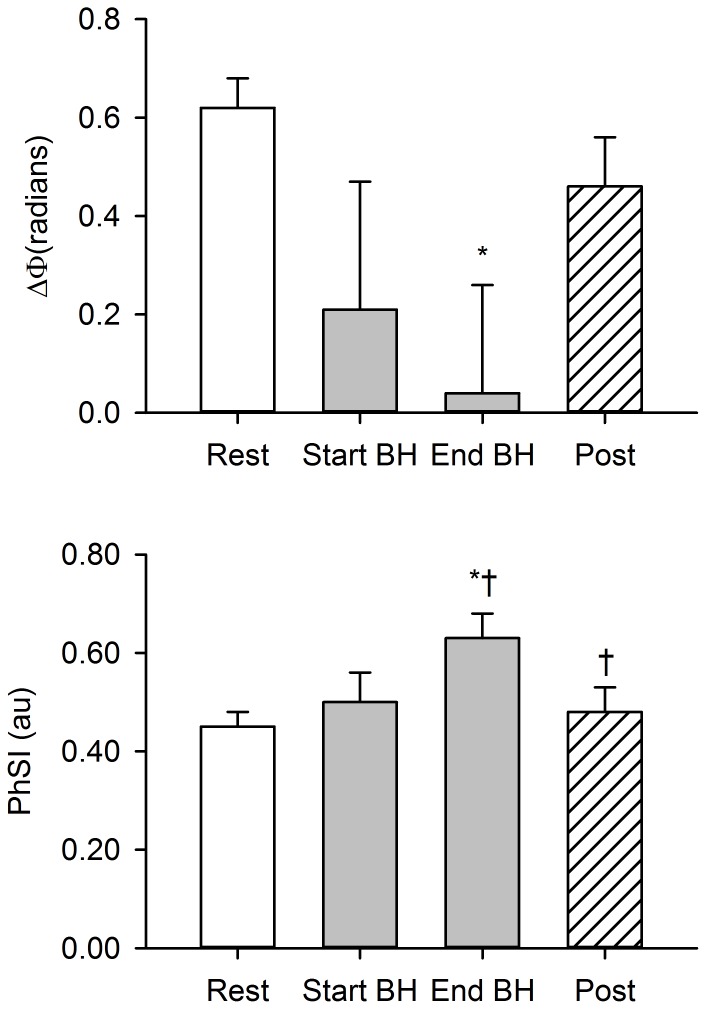
Group-averaged data for phase angle (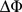) and phase synchronisation index (PhSI) during maximal voluntary apnoea. Bars are means ± SEM from 11 divers. BH: breath-hold. *P<0.05 compared with resting values. †*P*<0.05 compared with previous time-point.

**Figure 1** also displays the changes in 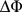 and PhSI during maximal apnoea for representative trained diver. **Figure 2** displays the group-averaged values for 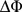 and PhSI obtained before, during and after maximal voluntary apnoea. PhSI had increased to values higher than resting values by the breakpoint of maximal voluntary apnoea (*P*<0.05). The mean change in PhSI from rest until the apnoea breakpoint (Δ) was 0.18±0.03. PhSI had returned to baseline by the end of the post-apnoea recovery period (*P*<0.05 compared to end of apnoea). The phase angle between MAP and CBFV (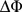) decreased below resting values during maximal apnoea (*P*<0.05), approaching values close to zero by the breakpoint.

**Figure 3:**
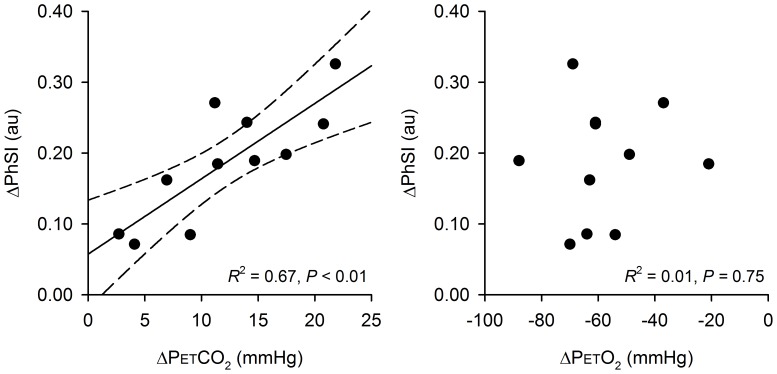
The influence of CO_2_ and O_2_ on the dynamic cerebral autoregulation during maximal voluntary apnoea. BH: breath-hold; PhSI: phase synchronisation index between the MAP and CBFV time series; PETCO_2_: end-tidal partial pressure of CO_2_; PETO_2_: end-tidal partial pressure of O_2_; Δ: absolute difference in magnitude between resting values and those observed at the breakpoint of apnoea. This figure demonstrates that those individuals who displayed the largest rise in PETCO_2_ also presented with the greatest impairment in dynamic cerebral autoregulation during apnoea. The fall in PETO_2_, and thus magnitude of arterial hypoxaemia, was not related to the impaired autoregulatory response observed during maximal apnoea in trained divers.

The absolute rise in PETCO_2_ from rest until the apnoea breakpoint was strongly, and positively correlated with the increase in PhSI over the same period of time (*R* ^2^ = 0.67, *P*<0.05, left-panel **Figure 3**). That is, the larger the rise in PETCO_2_ and, by extension, arterial tension of CO_2_, the greater the impairment in dCA during maximal voluntary apnoea. Interestingly, the magnitude of rise in PETO_2_ was not identified as significant correlate of the rise in PhSI throughout breath holding (*R* ^2^ = 0.01, *P* = 0.75, right-panel **Figure 3** **)**.

## Discussion

The novel findings of the present study were that: (i) the effectiveness of dCA is reduced during maximal voluntary apnoea in trained divers; and that (ii) this reduction was strongly related to the rise in PETCO_2_ during apnoea. Our data suggest that the ability of the cerebral vessels to autoregulate blood flow, and therefore to protect the brain against abrupt oscillations in driving pressure, is acutely impaired in divers performing maximal “dry” apnoea. These findings have important implications for the cerebrovascular health and safety of trained apnoea divers.

We report that dCA was acutely impaired during prolonged, voluntary apnoea. This inference is substantiated by two lines of evidence. First, PhSI had increased above resting values by the breakpoint of maximal apnoea. The rise in phase-coupling strength between MAP and CBFV indicates a progressive weakening of the autoregulatory response over time [22], [23]. Second, not only had 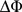 decreased during maximal apnoea, but its value approached zero by the breakpoint. It has been argued that when 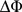 approximates zero, changes in MAP affect those in CBFV with no temporal delay, signifying autoregulatory failure [31], [32]. The present finding that maximal voluntary apnoea weakens dCA is consistent with observations made by others. For example, Przyby∤owski et al. [11] reported that approximately one-third of the cerebral blood flow response to brief apnoea (∼20 s) is mediated by increases in arterial blood pressure, indicating a partial failure of cerebral autoregulatory mechanisms during voluntary breath holding. More recently, Dineen et al. [33] demonstrated that the effectiveness of dCA is transiently reduced during voluntary apnoeas lasting ∼35 s. These findings, in conjunction with those of our own, supports the idea that voluntary breath holding acutely impairs dCA.

Many investigators have demonstrated that elevated arterial CO_2_ acutely impairs dCA during spontaneous breathing [1], [15]–[18], [34] – the precise mechanism(s) by which hypercapnia weakens the autoregulatory response is unclear. Nevertheless, our findings are in agreement with the above reports, insofar as the trained divers who displayed the largest rise in PETCO_2_ during maximal apnoea presented with the greatest impairment in dCA. It is well-established that isocapnic hypoxia impairs the cerebral autoregulatory response in awake, spontaneously breathing humans [19]–[21]. It is therefore surprising that we observed no direct relationship between the fall in PETO_2_ and the rise in PhSI during maximal apnoea. This finding does not necessarily obviate the thesis that arterial hypoxaemia contributes to the weakened autoregulatory response observed during maximal apnoea. Rather, it is possible that arterial O_2_ tension impacts on dCA in a manner not elucidated by the correlation analysis performed on our group of trained divers.

It must be appreciated that factors other than arterial blood gas tensions are known to modulate the effectiveness of dCA, these include: (i) sympathetic activity; (ii) metabolite production; and (iii) perivascular innervation [35]–[40]. Our data do not allow us to comment on the role that these factors played in disrupting autoregulatory processes during maximal apnoea. Further investigations are required to elucidate the precise mechanisms responsible for impairing dCA during prolonged, voluntary breath holding.

### Implications for the Cerebrovascular Health of Breath-hold Divers

The observation that PhSI had increased by the breakpoint of maximal apnoea begs the question: what is the impact of impaired autoregulation on cerebrovascular health during breath-hold diving? Certainly, there are numerous reports of serious brain damage and neurological impairment in habitual breath-hold divers [41]–[46]. However, the primary cause of these brain injuries is often attributed to cerebral arterial gas embolism, resulting from pulmonary barotrauma and/or shunting of venous gas emboli formed in peripheral tissues at depth. In a state of impaired dCA, the cerebral vessels behave more like a “pressure passive” system, wherein low to very-low frequency variations in arterial pressure (i.e., 0.01–0.15 Hz) are passively transmitted through the cerebrovasculature unabated, resulting in potentially severe fluctuations in cerebral blood flow and intracranial pressure. Following the above, one may speculate that arterial hypertension induced by apnoea [10], [13], [14], and concurrent impairment of cerebral autoregulation, may together promote the development of intracranial hypertension, and thus heighten the risk of cerebrovascular insult during breath-hold diving. Further research is warranted in order to determine whether impaired dCA during maximal breath holding leads to cerebrovascular insult in trained divers.

### Methodological Considerations

We used transcranial Doppler ultrasound of the right middle cerebral artery (MCA) to estimate bulk cerebral blood flow during maximal breath holding [47], [48]. This technique does not measure volumetric blood flow *per se*, rather it quantifies the velocity of blood passing through the insonated artery [49]. Changes in the radius of the insonated artery may therefore alter blood velocity, independent of volumetric flow through the MCA. Importantly, however, evidence suggests that MCA diameter is stable over a wide range of MAP and arterial blood gases [50], [51]. With this point in mind, we are confident that our values of cerebral blood flow-velocity is a valid marker of bulk cerebral blood flow. It must be acknowledged that only trained breath-hold divers were recruited in this study, and that all apnoeas were performed under “dry” laboratory conditions. Therefore, we caution that our findings may not apply to untrained/naïve participants, or to the conditions imposed on subjects when breath holding under water at depth. On this latter point, it was not feasible to use transcranial Doppler ultrasound to measure CBFV under water. Future studies may overcome this limitation via computational modelling of the pressure-flow characteristics of peripheral and cerebral vessels [52]–[54].

That the trained diver in **Figure 1** demonstrated a fall in PhSI within the first 60-s of apnoea suggests dCA transiently improved after the beginning of breath holding. This improved dCA may have been related to our observation that subjects, on average, were mildly hypocapnic immediately prior to breath holding. If this hypocapnia persisted throughout the first 60-s of apnoea, it is conceivable that the lowered arterial CO_2_ tension mediated an improvement in dCA – at least for the individual displayed in **Figure 1**. However, it was not feasible to measure PETCO_2_ during apnoeic efforts and, in turn, we cannot comment on whether arterial hypocapnia modulated dCA during the early phase of maximal apnoea. In relation to the above, it is of note that other investigators have reported that arterial hypercapnia does not develop to any great extent within the first 60 s of voluntary breath holding [55]–[57].

### Conclusions

The present study clearly demonstrates that the phase dynamics of cerebral autoregulation are acutely impaired during maximal “dry” apnoea in trained divers. Moreover, we provide evidence that the degree of impairment in dCA is related to the magnitude of rise in PETCO_2_ observed during apnoea. The potential for this impaired dCA to heighten the risk of cerebrovascular injury during breath-hold diving should be further explored.

## References

[1] Aaslid R, Lindegaard KF, Sorteberg W, Nornes H (1989) Cerebral autoregulation dynamics in humans. Stroke 20: 45–52. 2492126 10.1161/01.str.20.1.45

[2] Zhang R, Zuckerman JH, Giller CA, Levine BD (1998) Transfer function analysis of dynamic cerebral autoregulation in humans. American Journal of Physiology - Heart and Circulatory Physiology 274: H233–H241. 10.1152/ajpheart.1998.274.1.h233 9458872

[3] Dujić Z, Breskovic T, Bakovic D (2013) Breath-hold diving as a brain survival response. Translational Neuroscience 4: 302–313.

[4] Dujic Z, Breskovic T (2012) Impact of breath holding on cardiovascular respiratory and cerebrovascular health. Sports Medicine 42: 459–472. 22574634 10.2165/11599260-000000000-00000

[5] Ferris EB, Engel GL, Stevens CD, Webb J (1946) Voluntary Breathholding. Iii. The Relation of the Maximum Time of Breathholding to the Oxygen and Carbon Dioxide Tensions of Arterial Blood, with a Note on Its Clinical and Physiological Significance. Journal of Clinical Investigation 25: 734–743. 10.1172/JCI101757 PMC435613 16695367

[6] Klocke FJ, Rahn H (1959) Breath holding after breathing of oxygen. Journal of Applied Physiology 14: 689–693. 14409935 10.1152/jappl.1959.14.5.689

[7] Schneider EC (1930) Observations on holding the breath. American Journal of Physiology 94: 464–470.

[8] Andersson JPA, Linér MH, Jönsson H (2009) Increased serum levels of the brain damage marker S100B after apnea in trained breath-hold divers: a study including respiratory and cardiovascular observations. Journal of Applied Physiology 107: 809–815. 19574501 10.1152/japplphysiol.91434.2008

[9] Overgaard K, Friis S, Pedersen R, Lykkeboe G (2006) Influence of lung volume, glossopharyngeal inhalation and P ET O2 and P ET CO2 on apnea performance in trained breath-hold divers. European Journal of Applied Physiology 97: 158–164. 16525813 10.1007/s00421-006-0156-2

[10] Palada I, Obad A, Bakovic D, Valic Z, Ivancev V, et al (2007) Cerebral and peripheral hemodynamics and oxygenation during maximal dry breath-holds. Respiratory Physiology & Neurobiology 157: 374–381. 17363344 10.1016/j.resp.2007.02.002

[11] Przyby∤owski T, Bangash M-F, Reichmuth K, Morgan BJ, Skatrud JB, et al (2003) Mechanisms of the cerebrovascular response to apnoea in humans. The Journal of Physiology 548: 323–332. 12588894 10.1113/jphysiol.2002.029678 PMC2342799

[12] Kjeld T, Pott FC, Secher NH (2009) Facial immersion in cold water enhances cerebral blood velocity during breath-hold exercise in humans. Journal of Applied Physiology 106: 1243–1248. 19179653 10.1152/japplphysiol.90370.2008

[13] Heusser K, Dzamonja G, Tank J, Palada I, Valic Z, et al (2009) Cardiovascular Regulation During Apnea in Elite Divers. Hypertension 53: 719–724. 19255361 10.1161/HYPERTENSIONAHA.108.127530

[14] Andersson JPA, Linér MH, Rünow E, Schagatay EKA (2002) Diving response and arterial oxygen saturation during apnea and exercise in breath-hold divers. Journal of Applied Physiology 93: 882–886. 12183481 10.1152/japplphysiol.00863.2001

[15] Mitsis GD, Poulin MJ, Robbins PA, Marmarelis VZ (2004) Nonlinear modeling of the dynamic effects of arterial pressure and CO_2_ variations on cerebral blood flow in healthy humans. IEEE Transactions on Biomedical Engineering 51: 1932–1943. 15536895 10.1109/TBME.2004.834272

[16] Panerai RB, Deverson ST, Mahony P, Hayes P, Evans DH (1999) Effect of CO_2_ on dynamic cerebral autoregulation measurement. Physiological Measurement 20: 265. 10475580 10.1088/0967-3334/20/3/304

[17] Birch AA, Dirnhuber MJ, Hartley-Davies R, Iannotti F, Neil-Dwyer G (1995) Assessment of Autoregulation by Means of Periodic Changes in Blood Pressure. Stroke 26: 834–837. 7740576 10.1161/01.str.26.5.834

[18] Ursino M, Lodi CA (1998) Interaction among autoregulation, CO_2_ reactivity, and intracranial pressure: a mathematical model. American Journal of Physiology - Heart and Circulatory Physiology 274: H1715–H1728. 10.1152/ajpheart.1998.274.5.H1715 9612384

[19] Mardimae A, Balaban DY, Machina MA, Battisti-Charbonney A, Han JS, et al (2012) The interaction of carbon dioxide and hypoxia in the control of cerebral blood flow. Pflugers Arch 464: 345–351. 22961068 10.1007/s00424-012-1148-1

[20] Querido JS, Ainslie PN, Foster GE, Henderson WR, Halliwill JR, et al (2013) Dynamic cerebral autoregulation during and following acute hypoxia: role of carbon dioxide. Journal of Applied Physiology 114: 1183–1190. 23471947 10.1152/japplphysiol.00024.2013

[21] Ogoh S, Nakahara H, Ainslie PN, Miyamoto T (2010) The effect of oxygen on dynamic cerebral autoregulation: critical role of hypocapnia. Journal of Applied Physiology 108: 538–543. 20056845 10.1152/japplphysiol.01235.2009

[22] Latka M, Turalska M, Glaubic-Latka M, Kolodziej W, Latka D, et al (2005) Phase dynamics in cerebral autoregulation. American Journal of Physiology - Heart and Circulatory Physiology 289: H2272–2279. 16024579 10.1152/ajpheart.01307.2004

[23] Ocon AJ, Kulesa J, Clarke D, Taneja I, Medow MS, et al (2009) Increased phase synchronization and decreased cerebral autoregulation during fainting in the young. American Journal of Physiology - Heart and Circulatory Physiology 297: H2084–2095. 19820196 10.1152/ajpheart.00705.2009 PMC2793131

[24] Miller MR, Hankinson J, Brusasco V, Burgos F, Casaburi R, et al (2005) Standardisation of spirometry. European Respiratory Journal 26: 319–338. 16055882 10.1183/09031936.05.00034805

[25] O'Leary DD, Shoemaker JK, Edwards MR, Hughson RL (2004) Spontaneous beat-by-beat fluctuations of total peripheral and cerebrovascular resistance in response to tilt. American Journal of Physiology - Regulatory, Integrative and Comparative Physiology 287: R670–R679. 10.1152/ajpregu.00408.2003 15117726

[26] Rosenblum MG, Kurths J, Pikovsky A, Schafer C, Tass P, et al (1998) Synchronization in noisy systems and cardiorespiratory interaction. IEEE Engineering in Medicine and Biology Magazine 17: 46–53. 9824761 10.1109/51.731320

[27] Rosenblum MG, Cimponeriu L, Bezerianos A, Patzak A, Mrowka R (2002) Identification of coupling direction: application to cardiorespiratory interaction. Physical review E, Statistical, nonlinear, and soft matter physics 65: 041909. 10.1103/PhysRevE.65.041909 12005875

[28] Le Van Quyen M, Foucher J, Lachaux J, Rodriguez E, Lutz A, et al (2001) Comparison of Hilbert transform and wavelet methods for the analysis of neuronal synchrony. Journal of neuroscience methods 111: 83–98. 11595276 10.1016/s0165-0270(01)00372-7

[29] Quian Quiroga R, Kraskov A, Kreuz T, Grassberger P (2002) Performance of different synchronization measures in real data: a case study on electroencephalographic signals. Physical review E, Statistical, nonlinear, and soft matter physics 65: 041903. 10.1103/PhysRevE.65.041903 12005869

[30] Ocon AJ, Medow MS, Taneja I, Stewart JM (2011) Respiration drives phase synchronization between blood pressure and RR interval following loss of cardiovagal baroreflex during vasovagal syncope. American Journal of Physiology - Heart and Circulatory Physiology 300: H527–540. 21076019 10.1152/ajpheart.00257.2010 PMC3044066

[31] Diehl RR, Linden D, Lücke D, Berlit P (1995) Phase Relationship Between Cerebral Blood Flow Velocity and Blood Pressure: A Clinical Test of Autoregulation. Stroke 26: 1801–1804. 7570728 10.1161/01.str.26.10.1801

[32] Reinhard M, Roth M, Müller T, Guschlbauer B, Timmer J, et al (2004) Effect of Carotid Endarterectomy or Stenting on Impairment of Dynamic Cerebral Autoregulation. Stroke 35: 1381–1387. 15087557 10.1161/01.STR.0000127533.46914.31

[33] Dineen NE, Brodie FG, Robinson TG, Panerai RB (2010) Continuous estimates of dynamic cerebral autoregulation during transient hypocapnia and hypercapnia. Journal of Applied Physiology 108: 604–613. 20035062 10.1152/japplphysiol.01157.2009 PMC2838633

[34] Maggio P, Salinet AS, Panerai RB, Robinson TG (2013) Does hypercapnia-induced impairment of cerebral autoregulation affect neurovascular coupling? A functional TCD study. Journal of Applied Physiology 115: 491–497. 23743398 10.1152/japplphysiol.00327.2013 PMC3742941

[35] ter Laan M, van Dijk JMC, Elting JWJ, Staal MJ, Absalom AR (2013) Sympathetic regulation of cerebral blood flow in humans: a review. British Journal of Anaesthesia 111: 361–367. 23616589 10.1093/bja/aet122

[36] Zhang R, Zuckerman JH, Iwasaki K, Wilson TE, Crandall CG, et al (2002) Autonomic Neural Control of Dynamic Cerebral Autoregulation in Humans. Circulation 106: 1814–1820. 12356635 10.1161/01.cir.0000031798.07790.fe

[37] Mitsis GD, Zhang R, Levine BD, Tzanalaridou E, Katritsis DG, et al (2009) Autonomic neural control of cerebral hemodynamics. IEEE Engineering in Medicine and Biology Magazine 28: 54–62. 19914889 10.1109/MEMB.2009.934908 PMC2917725

[38] Hamel E (2006) Perivascular nerves and the regulation of cerebrovascular tone. Journal of Applied Physiology 100: 1059–1064. 16467392 10.1152/japplphysiol.00954.2005

[39] Peterson EC, Wang Z, Britz G (2011) Regulation of cerebral blood flow. International Journal of Vascular Medicine 2011: 823525. 21808738 10.1155/2011/823525 PMC3144666

[40] Toda N, Ayajiki K, Okamura T (2009) Cerebral Blood Flow Regulation by Nitric Oxide: Recent Advances. Pharmacological Reviews 61: 62–97. 19293146 10.1124/pr.108.000547

[41] Tamaki H, Kohshi K, Sajima S, Takeyama J, Nakamura T, et al (2010) Repetitive breath-hold diving causes serious brain injury. Undersea & hyperbaric medicine 37: 7–11. 20369648

[42] Melamed Y, Shupak A, Bitterman H (1992) Medical problems associated with underwater diving. New England Journal of Medicine 326: 30–35. 1727063 10.1056/NEJM199201023260105

[43] Kohshi K, Katoh T, Abe H, Okudera T (2000) Neurological accidents caused by repetitive breath-hold dives: two case reports. Journal of the Neurological Sciences 178: 66–69. 11018252 10.1016/s0022-510x(00)00360-9

[44] Kohshi K, Wong RM, Abe H, Katoh T, Okudera T, et al (2005) Neurological manifestations in Japanese Ama divers. Undersea & hyperbaric medicine 32: 11–20. 15796310

[45] Gempp E, Blatteau JE (2006) Neurological disorders after repetitive breath-hold diving. Aviation Space and Environmental Medicine 77: 971–973. 16964749

[46] Ridgway L, McFarland K (2006) Apnea diving: long-term neurocognitive sequelae of repeated hypoxemia. The Clinical neuropsychologist 20: 160–176. 16410228 10.1080/13854040590947407

[47] Dahl A, Lindegaard KF, Russell D, Nyberg-Hansen R, Rootwelt K, et al (1992) A comparison of transcranial Doppler and cerebral blood flow studies to assess cerebral vasoreactivity. Stroke 23: 15–19. 1731414 10.1161/01.str.23.1.15

[48] Cross TJ, Kavanagh JJ, Breskovic T, Zubin Maslov P, Lojpur M, et al (2013) The Effects of Involuntary Respiratory Contractions on Cerebral Blood Flow during Maximal Apnoea in Trained Divers. PLoS One 8: e66950. 23840561 10.1371/journal.pone.0066950 PMC3694127

[49] Kontos HA (1989) Validity of cerebral arterial blood flow calculations from velocity measurements. Stroke 20: 1–3. 2911822 10.1161/01.str.20.1.1

[50] Poulin MJ, Robbins PA (1996) Indexes of Flow and Cross-sectional Area of the Middle Cerebral Artery Using Doppler Ultrasound During Hypoxia and Hypercapnia in Humans. Stroke 27: 2244–2250. 8969788 10.1161/01.str.27.12.2244

[51] Serrador JM, Picot PA, Rutt BK, Shoemaker JK, Bondar RL (2000) MRI Measures of Middle Cerebral Artery Diameter in Conscious Humans During Simulated Orthostasis. Stroke 31: 1672–1678. 10884472 10.1161/01.str.31.7.1672

[52] Wong KKL, Mazumdar J, Pincombe B, Worthley SG, Sanders P, et al (2006) Theoretical modeling of micro-scale biological phenomena in human coronary arteries. Medical & biological engineering & computing 44: 971–982. 17048027 10.1007/s11517-006-0113-6

[53] Wong KKL, Tu J, Mazumdar J, Abbott D (2010) Modelling of blood flow resistance for an atherosclerotic artery with multiple stenoses and poststenotic dilatations. ANZIAM Journal 51: C66–C82.

[54] Wong KKL, Tu J, Sun Z, Dissanayake D (2013) Methods in Research and Development of Biomedical Devices. Singapore: World Scientific Publishing Co.

[55] Hong SK, Lin YC, Lally DA, Yim BJB, Kominami N, et al. (1971) Alveolar Gas Exchanges and Cardiovascular Functions during Breath Holding with Air. Journal of Applied Physiology 30: 540–&. 10.1152/jappl.1971.30.4.540 4929469

[56] Ferretti G, Costa M, Ferrigno M, Grassi B, Marconi C, et al (1991) Alveolar gas composition and exchange during deep breath-hold diving and dry breath holds in elite divers. Journal of Applied Physiology 70: 794–802. 1902459 10.1152/jappl.1991.70.2.794

[57] Liner MH, Ferrigno M, Lundgren CE (1993) Alveolar gas exchange during simulated breath-hold diving to 20 m. Undersea Hyperb Med 20: 27–38. 8471957

